# Role of Gum Chewing in Post-operative Gut Motility After Caesarean Section

**DOI:** 10.7759/cureus.94520

**Published:** 2025-10-14

**Authors:** Mahrukh Ehsan, Sehrish Sabir, Abida Ashraf, Mariam Zahir

**Affiliations:** 1 Obstetrics and Gynecology, Peterborough City Hospital, Peterborough, GBR; 2 Obstetrics and Gynecology, Al Azhar Hospital, Riyadh, SAU; 3 Obstetrics and Gynecology, Social Security Teaching Hospital, Lahore, PAK; 4 Department of Obstetrics and Gynecology, Benazir Bhutto Hospital Rawalpindi, Rawalpindi, PAK

**Keywords:** c-section, gum chewing, patients, postoperative ileus, recovery support

## Abstract

Introduction: Postoperative ileus (POI) is a common complication following abdominal surgeries, including cesarean sections (C-sections), and can significantly delay recovery. Although there is no specific treatment for POI, strategies like nasogastric suction, early feeding, intravenous fluids, and local analgesics can reduce the risk of paralytic ileus.

Objectives: The objective of this study was to compare the effect of gum chewing on postoperative recovery of bowel function following C-section, specifically focusing on the time to first bowel sounds and the time to first defecation.

Methodology: This comparative observational study was conducted at the Department of Obstetrics and Gynaecology, Benazir Bhutto Hospital, Rawalpindi, from 5th August 2016 to 4th February 2017. A total of 100 women, aged 18-44 years, undergoing elective C-sections were selected. Exclusion criteria included a history of drug consumption (especially opioids), peritonitis, and previous abdominal surgery. Participants were enrolled using purposive sampling, with 50 patients in the experimental group (gum chewing) and 50 in the control group (non-gum chewing). In the experimental group, patients chewed two sticks of commercially available sugar-free gum three times daily for 15-20 minutes starting immediately after surgery. Both groups were followed until discharge, and the time to first bowel sounds and first defecation were recorded.

Results: The mean age of patients in the experimental group was 29.52 ± 6.70 years, and in the control group, it was 29.88 ± 7.40 years. The mean gestational age was 39.63 ± 1.58 weeks. The mean time to first bowel sounds in the experimental group was 18.06 ± 5.11 hours, compared to 24.42 ± 8.33 hours in the control group (p-value = 0.0001). The mean time to first defecation in the experimental group was 31.5 ± 4.85 hours, while in the control group, it was 39.52 ± 5.61 hours (p-value = 0.0001).

Conclusion: The study concluded that gum chewing significantly reduces the time to first bowel sounds and the time to first defecation in postoperative C-section patients, suggesting that this simple intervention accelerates recovery from postoperative ileus.

## Introduction

Cesarean section (C-section) remains one of the most frequently performed major surgical procedures in obstetrics worldwide. Despite advances in surgical and anesthetic techniques, postoperative complications such as decreased bowel motility and paralytic ileus continue to pose significant challenges to maternal recovery and healthcare efficiency [1]. Postoperative ileus (POI) refers to the temporary cessation of coordinated bowel motility following abdominal surgery, resulting in delayed passage of flatus or stool, abdominal distension, nausea, vomiting, and intolerance to oral intake [2]. These symptoms contribute to patient discomfort, prolonged hospital stay, and delayed initiation of breastfeeding. The pathogenesis of POI is multifactorial, involving activation of inhibitory neural reflexes, local inflammatory responses, and altered autonomic balance. Surgical stress leads to sympathetic overactivity and suppression of parasympathetic (vagal) stimulation, reducing acetylcholine release in the gut and thereby impairing peristalsis [3]. Additional contributors include intraoperative bowel handling, electrolyte disturbances, use of anesthetic agents, and postoperative opioid analgesics [4]. While POI is generally self-limiting, it remains a preventable cause of morbidity that delays postoperative recovery. Conventional management is primarily supportive; early ambulation, intravenous hydration, electrolyte correction, and nasogastric decompression are standard measures. However, these interventions have a limited impact on the early restoration of bowel activity [5]. Consequently, attention has shifted toward non-pharmacological strategies that mimic physiological feeding responses to stimulate gastrointestinal motility. One such approach is sham feeding, particularly chewing gum, which imitates the act of eating and stimulates the cephalic-vagal pathway, promoting the secretion of gastrointestinal hormones and the initiation of peristaltic reflexes [6,7].

Several randomized and observational studies have shown that postoperative gum chewing accelerates bowel function recovery in patients undergoing abdominal and gynecological surgeries, including cesarean delivery [8,9]. The intervention is safe, inexpensive, and easily applicable in resource-limited healthcare settings. Incorporating such a strategy into standard postoperative protocols could not only shorten hospital stay and improve maternal comfort but also optimize bed utilization and reduce healthcare costs. The implementation of gum chewing or otherwise sham feeding in our environment can be very beneficial [10]. It improves postoperative rehabilitation and early ambulation, shortens hospitalization, minimizes the risk of deep vein thrombosis and frees the beds to be used by new patients. The study justification is that it will determine the effectiveness of chewing gum that can facilitate the improved management of obstetric and gynecological patients, as well as improved postoperative recovery and shorter length of hospital stay [11].

The objective of the study was to compare the effect of gum chewing on postoperative recovery of bowel function after C-section, specifically in terms of time to the first bowel sounds and time to the first defecation.

## Materials and methods

This comparative observational study was conducted at the Department of Obstetrics and Gynaecology, Benazir Bhutto Hospital, Rawalpindi, from August 5, 2016, to February 4, 2017. Data were collected from 100 patients (50 in each group). The sample size for this study was calculated using the WHO sample size calculator, based on a 5% level of confidence and a 90% power of the test. The calculation assumed a population mean of 21.5% and a test value of 26%, with a pooled standard deviation of 8 [https://www.who.int/tools/sample-size-calculator]. Participants were included in the study if they met the following criteria: term pregnancy, undergoing elective lower segment cesarean section via Pfannenstiel incision, under regional anesthesia, and aged between 18 and 44 years. Exclusion criteria included a history of drug use, particularly opioids, or the presence of water and electrolyte disturbances, pancreatitis, or peritonitis. Patients with a history of any abdominal surgery other than a previous C-section, those unwilling to cooperate, or those who experienced intraoperative or postoperative complications were also excluded. Additional exclusions included women with diabetes mellitus, muscular or neurological disorders, pre-eclampsia, or those unable to chew gum.

Data collection procedure

The study was conducted after approval from the ethical committee of Rawalpindi Medical College (RMC, approval RMC/ERC/2016/OBS-GYN-017). All patients fulfilling the inclusion criteria were included in the study after obtaining informed consent. A sociodemographic data sheet was filled out for each subject. Protocols for the administration of chewing gum, auscultation of bowel sounds, and the time of passage of the first stool were recorded. A total of 100 patients were enrolled through purposive sampling, with 50 patients in the experimental group (gum chewing) and 50 patients in the control group. In the experimental group, patients were instructed to chew two sticks of commercially available sugar-free gum three times a day for 15-20 minutes each time, starting immediately after surgery. All patients were followed up until discharge from the hospital. The primary outcomes were the time to return of bowel sounds and the time to first defecation, both measured in hours after surgery. 

Data analysis procedure

Data was analyzed using SPSS version 20 (IBM Corp., Armonk, NY, USA). For qualitative variables, frequency and percentages were assessed. For quantitative variables like age and parity, means and standard deviations were calculated. Data was presented in tables. To assess the statistically significant difference in the reduction of postoperative ileus between the two groups, an independent t-test was performed at a 5% level of significance. Effect modifiers such as age, gestational age, and parity were controlled through stratification. Post-stratification, an independent sample t-test was applied. A p-value ≤ 0.05 was considered statistically significant.

## Results

In the experimental group, 31 (62%) were aged 18-30 years, and in the control group, 30 (60%) fell in the same age range, with a mean age around 29.7 ± 7.03 years across both groups. Gestational age was 37-39 weeks in 27 (54%) of experimental and 28 (56%) of control participants. Parity distribution showed that 74% of women in the gum-chewing group and 76% in the control group had three or fewer previous deliveries, with mean parity values of 2.36 ± 1.56 and 2.42 ± 1.38, respectively (Table 1).

**Table 1:** Demographic Characteristics for Both Groups (n = 100)

Characteristic	Experimental (n = 50)	Control (n = 50)	Total (n = 100)
Age (years)			
18-30	31 (62.0%)	30 (60.0%)	61 (61.0%)
31-44	19 (38.0%)	20 (40.0%)	39 (39.0%)
Mean ± SD	29.52 ± 6.70	29.88 ± 7.40	29.70 ± 7.03
Gestational Age (weeks)			
37-39	27 (54.0%)	28 (56.0%)	55 (55.0%)
40-42	23 (46.0%)	22 (44.0%)	45 (45.0%)
Mean ± SD	39.44 ± 1.28	39.12 ± 1.09	39.63 ± 1.58
Parity			
≤3	37 (74.0%)	38 (76.0%)	75 (75.0%)
>3	13 (26.0%)	12 (24.0%)	25 (25.0%)
Mean ± SD	2.36 ± 1.56	2.42 ± 1.38	2.51 ± 1.49

The mean time to first bowel sounds was 18.06 ± 5.11 hours in the experimental group compared to 24.42 ± 8.33 hours in the control group (p = 0.0001). Similarly, the mean time to first defecation was reduced in the experimental group at 31.5 ± 4.85 hours versus 39.52 ± 5.61 hours in the control group, showing a statistically significant difference (p = 0.0001) (Table 2).

**Table 2:** Time to First Bowel Sounds and Time to First Defecation for Both Groups P-values were calculated using the Independent Samples t-test to assess the significance of differences between the experimental (gum-chewing) and control groups.

Measure	Experimental (n = 50)	Control (n = 50)	P-value
Time to First Bowel Sounds (hours)	18.06 ± 5.11	24.42 ± 8.33	0.0001
Time to First Defecation (hours)	31.5 ± 4.85	39.52 ± 5.61	0.0001

Among women aged 18-30 years, bowel sounds returned in 17.84 ± 5.25 hours for the experimental group and 24.60 ± 8.61 hours for controls (p = 0.0006). Across gestational age and parity subgroups, similar trends were noted with statistically significant differences, such as 18.32 ± 4.89 hours versus 23.78 ± 9.32 hours in the 37-39 weeks gestation group (p = 0.0092), and 17.85 ± 5.09 versus 23.33 ± 9.39 hours among parity ≤3 patients (p = 0.0026) (Table 3).

**Table 3:** Stratification of Time to First Bowel Sounds with Respect to Age, Gestational Age, and Parity P-values were calculated using the Independent Samples t-test to compare the experimental and control groups within each stratified subgroup.

Characteristic	Experimental (n = 50)	Control (n = 50)	P-value
Age (years)			
18-30	17.84 ± 5.25	24.60 ± 8.61	0.0006
31-44	18.42 ± 5.00	24.15 ± 9.60	0.0256
Gestational Age (weeks)			
37-39	18.32 ± 4.89	23.78 ± 9.32	0.0092
40-42	18.65 ± 5.33	24.68 ± 9.48	0.0122
Parity			
≤3	17.85 ± 5.09	23.33 ± 9.39	0.0026
>3	19.01 ± 5.24	25.45 ± 8.88	0.0427

Participants aged 18-30 years had first defecation at 31.45 ± 4.99 hours in the experimental group compared to 39.87 ± 5.76 hours in the control group (p = 0.0001). Similar reductions were seen across gestational age groups, where 37-39 weeks gestation patients had defecation at 31.02 ± 4.87 versus 39.43 ± 5.66 hours, and for parity ≤3, it was 31.12 ± 4.67 versus 39.23 ± 5.35 hours, all showing highly significant p-values (Table 4).

**Table 4:** Stratification of Time to First Defecation with Respect to Age, Gestational Age, and Parity Independent samples t-test was used to analyze p-values.

Characteristic	Experimental (n = 50)	Control (n = 50)	P-value
Age (years)			
18-30	31.45 ± 4.99	39.87 ± 5.76	0.0001
31-44	31.58 ± 4.75	39.00 ± 5.49	0.0001
Gestational Age (weeks)			
37-39	31.02 ± 4.87	39.43 ± 5.66	0.0001
40-42	31.53 ± 4.92	39.04 ± 5.54	0.0001
Parity			
≤3	31.12 ± 4.67	39.23 ± 5.35	0.0001
>3	31.68 ± 4.87	39.59 ± 5.81	0.0001

## Discussion

The results of this research point at the evident relationship between the gum-chewing habit and accelerated postoperative gastric motility after caesarean section. Experimental group members, where gum-chewing took place, to first bowel sounds and first defecation times that were also significantly shorter than they were in the control group. In particular, the average time to first bowel sounds became shorter in the experimental community compared with the control one, 24.42 +/- 8.33 hours to 18.06 +/- 5.11 hours, according to the mean time to first defecation, which was measured at 39.52 +/- 5.61 hours and 31.5 +/- 4.85 hours, respectively [12]. These effects did not vary between the various different ages, gestational ages, and parities, and this is supported by the stratified analysis where all p-values reached a statistical statistical significance. The findings are in agreement with the early studies that gum chewing excites cephalic-vagal paths that enhance the motility of the gastrointestinal tract in the same mechanisms as sham feeding [13]. The positive effect in the subgroups notes the gum chewing as a potentially universal, non-pharmacological intervention that may be adopted as a routine measure in the post-cesarean recovery practices. But this difference in magnitude indicates the clinical importance of decreasing postoperative discomfort, lowering risks of ileus and potentially shortening length of hospital stay [14].

Ledari et al. (2012) investigated it and demonstrated how gum chewing can shorten the average time to first bowel movement of women following an elective cesarean section, in the experimental group (7.4 +/- 1.71 hours) than in the control group (15.7 +/- 3.44 hours) [15]. Nevertheless, a study by Morais et al. (2016) conducted a systematic review did not produce any statistically significant difference on the time to bowel movement between the gum-chewing and control groups (7.09 hours, 95% CI -9.27 to -4.91 hours) [16]. The difference can be explained by sampling and surgical condition. The primary limitations of this study include its relatively small sample size of 100 participants, which may limit the generalizability of the findings to a broader population. Additionally, the study was conducted in a single center, introducing potential institutional bias. Factors such as variations in surgical technique, anesthesia type, and post-operative care protocols were not controlled, which could influence gastrointestinal motility outcomes. Patient adherence to the gum-chewing protocol was based on self-reporting, posing a risk of reporting bias.

## Conclusions

It is concluded that postoperative gum chewing significantly enhances gastrointestinal recovery in women undergoing cesarean section. Patients who chewed gum demonstrated an earlier return of bowel sounds and quicker passage of stool compared to those who did not, reflecting improved bowel motility and reduced postoperative discomfort. Given its simplicity, safety, and cost-effectiveness, gum chewing represents a valuable non-pharmacological intervention that can be easily incorporated into standard postoperative care to promote faster recovery, facilitate early ambulation, shorten hospital stay, and improve overall maternal outcomes after cesarean delivery.
